# Amylase-Dependent Regulation of Glucose Metabolism and Insulin/Glucagon Secretion in the Streptozotocin-Induced Diabetic Pig Model and in a Rat Pancreatic Beta-Cell Line, BRIN-BD11

**DOI:** 10.1155/2020/2148740

**Published:** 2020-11-17

**Authors:** Kateryna Pierzynowska, Stina Oredsson, Stefan Pierzynowski

**Affiliations:** ^1^Department of Animal Physiology, The Kielanowski Institute of Animal Nutrition and Physiology Polish Academy of Sciences, Instytucka 3, 05110 Jabłonna, Poland; ^2^Department of Biology, Lund University, Sölvegatan 35, 22362 Lund, Sweden; ^3^SGPlus-Group, Alfågelgränden 24, 23132 Trelleborg, Sweden; ^4^Department of Biology, Institute Rural Medicine, Jaczewskiego 2, 20950 Lublin, Poland

## Abstract

The current study was aimed at highlighting the role of blood pancreatic amylase in the regulation of glucose homeostasis and insulin secretion in a porcine model of streptozotocin- (STZ-) induced diabetes and in a rat pancreatic beta-cell line, BRIN-BD11. Blood glucose, plasma insulin, and glucagon levels were measured following a duodenal glucose tolerance test (IDGTT), in four pigs with STZ-induced type 2 diabetes (T2D pigs) and in four pigs with STZ-induced type 1 diabetes (T1D pigs). Four intact pigs were used as the control group. The effect of amylase supplementation on both acute and chronic insulin secretion was determined in a BRIN-BD11 cell line. The amylase infusion had no effect on the glucose utilization curve or glucagon levels in the healthy pigs. However, a significant lowering of insulin release was observed in healthy pigs treated with amylase. In the T2D pigs, the glucose utilization curve was significantly lowered in the presence of amylase, while the insulin response curve remained unchanged. Amylase also significantly increased glucagon release during the IDGTT in the T2D and T1D pigs, by between 2- and 4-fold. Amylase did not affect the glucose utilization curve in the T1D pigs. Amylase supplementation significantly decreased both acute and chronic insulin secretion in the BRIN-BD11 cells. These data confirm our previous observations and demonstrate the participation of pancreatic amylase in glucose absorption/utilization. Moreover, the present study clearly highlights the direct impact of pancreatic blood amylase on insulin secretion from pancreatic beta-cells and its interactions with insulin and glucagon secretion in a porcine model.

## 1. Introduction

Diabetes is the first, noninfectious disease recognized by the United Nations as an epidemic of the XXI century [1]. Approximately 87-91% of diabetes cases are type 2 diabetes [2]. The first WHO global report on diabetes demonstrates that the number of adults living with diabetes has almost quadrupled since 1980 to 422 million adults. This dramatic rise is largely due to the rise in type 2 diabetes [3].

Due to similarities in the morphology and physiology of the gastrointestinal systems, enzymatic and hormonal factors, transit times of ingesta, and digestive efficiencies, the diabetic pig model is currently becoming a popular large animal model of human disease, used to investigate endocrinology and metabolism [4–6]. Streptozotocin (STZ), a naturally occurring antineoplastic agent, acting with selective toxicity to the beta-cells of the pancreas, has frequently been used to induce insulin-deficient diabetes in many species [7–13]. Both type 1-like [4, 12] and type 2-like [14, 15] diabetes mellitus can be induced, depending on the dose and manner in which STZ is administered. Rapid intravenous injection of STZ, at doses of around 100 mg/kg or higher, produces insulin-dependent diabetes in pigs within days after STZ administration [9, 12]. Lower doses of STZ generally result in a transient or absent diabetic reaction in pigs [9, 16].

High endogenous serum amylase activity was recently shown to be associated with improved glucose homeostasis [17]. Conversely, low serum amylase is associated with an increased risk/prevalence of metabolic syndrome [18, 19] and raised BMI, as well as insulin resistance and decreased insulin levels [20]. A study by Nakajima and Magee in 1970 showed that pancreatic exocrine secretion, especially amylase secretion, was significantly inhibited following a rapid i.v. infusion of a 40% glucose solution [21], indicating a role for amylase in the maintenance of glucose homeostasis and possibly in obesity development. Normal serum amylase activity is strongly positively associated with the integrated function of the islet beta-cells, while low serum amylase levels are associated with impaired insulin secretion and sensitivity [22]. The results obtained from our own studies demonstrate that an intravenous infusion of pancreatic amylase is able to regulate the glucose-stimulated insulin response in young healthy pigs, as well as in pigs after duodenal-jejunal bypass surgery [23].

The present study was conducted to elucidate the effects of pancreatic amylase on plasma insulin, glucagon, and glucose levels during an intraduodenal glucose tolerance test (IDGTT), in healthy pigs and pigs with STZ-induced type 1-like and type 2-like diabetes mellitus, and investigate the possible direct effects of amylase on pancreatic beta-cells in the well-characterised, rat clonal, insulin-secreting beta-cell line, BRIN-BD11 [24].

## 2. Research Design and Methods

The present study was carried out in strict accordance with the recommendations in the Guide for the Care and Use of Laboratory Animals of the National Institutes of Health. All experimental procedures were approved by the Local Ethics Committee on Animal Experimentation of the Warsaw University of Life Sciences, Poland (Approval No. WAW2/15/2017). All efforts were made to minimize animal suffering during experimental procedures.

### 2.1. Animals

The experiment was performed on crossbred ((Polish Landrace × Yorkshire) × Hampshire)) pigs (*Sus scrofa domesticus*). Twelve male pigs, aged 10 ± 2 weeks and weighing 12 ± 3 kg at the start of experiment, were used in the study. The pigs were maintained on a 12-hour day-night cycle, with lights on from 06:00-18:00. The pigs were housed in individual cages. The cages were equipped with a feeding trough, drinking nipple, and constant heating lamp (150 W). The pigs were allowed to move freely within their cages and had visual contact with each other. The pigs were fed a standard cereal-based feed (“Morawski,” Żurawia, Poland) corresponding to 4% of their body weight daily, half the ration was given in the morning, between 08:00 and 09:00, and the other half in the afternoon, between 16:00 and 16:30. The pigs had free access to water for the duration of the experiment.

### 2.2. Surgery

After a five-day adaptation period, pigs were subjected to surgery to insert a duodenal fistula and jugular vein catheter for glucose enteral infusion and for blood sampling and STZ injection, respectively. Prior to surgery, all pigs were fasted for 24 hours and offered only water. The day after surgery, the pigs were given 25% of the food they consumed prior to surgery, and their postoperative food intake was established on the same level as before surgery.

Pigs were sedated using azaperone (Stresnil, LEO, Helsingborg, Sweden) at 4 mg/kg bw, i.m., then washed using surgical soap, and their abdominal and jugular regions were shaved. The pigs were then anaesthetised using 0.5–1.5% air mixture of Fluothane (Zeneca, Gothenburg, Sweden) and O_2_ as a carrier gas, at approximately 0.5–1 l/min in a close-circuit respiratory system (Komesaroff Medical Developments, Melbourne, Australia). Surgical anaesthesia was indicated by the lack of a corneal reflex. Thereafter, the pigs were intubated and placed on the surgical table and the site of the operation was disinfected with iodine solution and 70% ethanol. The surgery was performed under aseptic conditions. A 10–15 cm long incision was made posterior to the sternum, along the *linea alba*. Once the duodenum was located, a duodenal fistula was placed just after the entrance of pancreatic duct and secured using two silk sutures (Silk 0-2 Ethicon, Johnson and Johnson). The fistula was exteriorized through the skin and muscles on the right flank, in the middle of the abdominal cavity, just under the ribs. The abdomen was then stitched up using three layers of sutures, absorbable sutures for the muscle layers/pleura and nonabsorbable sutures for the skin. The jugular catheter was inserted into the *anterior vena cava*, via the right jugular vein.

Postoperative pain was prevented by the administration of buprenorphine (Temgesic®, Schering-Plough AB, 0.01 mg/kg bw, intramuscularly). Ampicillin (Doktacillin, Astra Läkemedel, Södertälje, Sweden) was administrated i.v. (15 mg/kg bw) for three days after surgery.

### 2.3. Induction of Diabetes and Experimental Design

**Figure 1:**
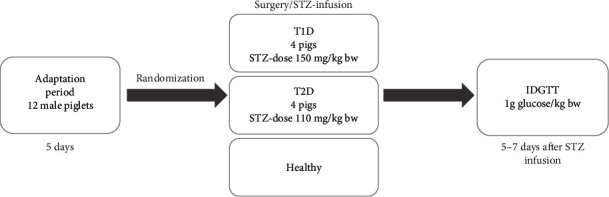
Schematic representation of the *in vivo* study design. IDGTT: intraduodenal glucose tolerance test; T1D: STZ-induced type 1-like diabetes mellitus; T2D: STZ-induced type 2-like diabetes mellitus.

A schematic representation of the study design is shown in Figure 1. Twelve male pigs were randomized into three groups: T1D (*n* = 4), pigs with STZ-induced type 1-like diabetes mellitus, which were administered STZ at 150 mg/kg bw; T2D (*n* = 4), pigs with STZ-induced type 2-like diabetes mellitus, which were administered STZ at 110 mg/kg bw; and the healthy (*n* = 4) pigs, which consisted of pigs which underwent surgical procedures but were not administered any STZ. Sample size was estimated using G∗Power software, version 3.1.9.4 [25] for a one-way ANOVA at *α* = 0.05 with 95% power, assuming *f*(effectsize) = 2 and SD = 50, for three study groups.

Type 1-like and type 2-like diabetes mellitus were induced by rapid (within 5 minutes), single intravenous infusion of STZ (S0130, Sigma-Aldrich Sweden AB, Stockholm, Sweden). STZ was dissolved in sterile disodium citrate buffer (0.08 g/ml) and administrated to the pigs via the jugular vein catheters. STZ (110 or 150 mg/kg bw for development of type 2-like and type 1-like diabetes mellitus, respectively) was infused at the end of the surgery, while the pigs were still under general anaesthesia. After induction of diabetes, fasting blood glucose levels were measured twice a day prior to administration of food, using a glucometer and test strips (Accu-Chek Aviva, Roche Diagnostics, Germany). Preprandial glucose levels 6-10 mmol/l were recognized as moderate hyperglycemia and the onset of type 2-like diabetes mellitus, while preprandial glucose levels over 10 mmol/l were recognized as hyperglycemia and the onset of type 1-like diabetes mellitus [15].

All twelve pigs were used to assess the effects of an intravenous amylase infusion on the outcome of IDGTT on days 5-7 after STZ infusion. A sterile solution of microbial (*Aspergillus oryzae*) pancreatic-like amylase (Amano Enzyme, Elgin, IL, USA) in 0.9% NaCl, 4000 U/pig, was administered twice via the jugular vein catheter just after first blood sampling at -60 minutes and then at 1 minute prior to performing the IDGTT. The amount of amylase infused was chosen based on previous experience [23], considering that the physiological concentration of amylase in interstitial fluid surrounding the pancreatic islets should be between 10 and 100 times higher than that in peripheral blood (400-500 U/l). Thus, to increase the amylase level in pancreatic islet circulation, we needed to reach an amylase level of between 4000 and 8000 U/l in the peripheral blood. For the IDGTT, glucose was administered as a 50% solution (1 g glucose/kg bw) via the duodenal fistula, which was flushed with 8 ml of 0.9% saline solution immediately thereafter. Each pig underwent the IDGTT twice, with and without (negative control) the intravenous amylase infusion, following a Latin square design. At the end of the study, all pigs were euthanized by an i.v. injection of an overdose of pentobarbital sodium (Allfatal Vet. Omnidea, Stockholm, Sweden, 100 mg/kg bw).

### 2.4. Blood Collection and Analysis

Blood samples were collected via the jugular vein catheter one hour and then again at one minute prior to glucose infusion and then at 5, 15, 30, 45, 60, and 120 minutes after the glucose infusion into the duodenum and transferred to BD Vacutainer® glass Aprotinine K_3_EDTA tubes (BD Diagnostics, New Jersey, USA). The blood samples were immediately placed on ice before they were centrifuged at 3000 × g for 15 minutes at 4°C, and plasma was separated and stored at -80°C until further analysis. Blood glucose concentrations were measured directly following blood sampling using a glucometer and test strips (Accu-Chek Aviva, Roche Diagnostics, Germany).

Plasma insulin and glucagon concentrations were measured using porcine insulin and porcine glucagon ELISA kits (cat nos. 10-1200-01 and 10-1281-01, respectively, Mercodia, Uppsala, Sweden), according to the manufacturer's instructions. Plasma amylolytic activity was analyzed using ethylidene-pNP-G7 (4,6-ethylidenep-nitrophenyl-alpha, D-maltoheptaoside) as the substrate, according to the manufacturer's instructions (Infinity Amylase Liquid Stable Reagent; Thermo Fisher Scientific, Middletown, Virginia, USA), with modifications for a microtiter plate reader.

### 2.5. Cell Culture

The rat clonal, insulin-secreting, BRIN-BD11 pancreatic beta-cell line (cat no. 10033003-1VL, Sigma-Aldrich, St. Louis, MO, USA) was used in this study [24]. Cells were routinely cultured in T75 flasks, with RPMI-1640 medium supplemented with 10% (*v*/*v*) fetal bovine serum, antibiotics (100 U/ml penicillin and 0.1 mg/ml streptomycin), and 2 mM L-glutamine, at 37°C in a humidified atmosphere of 5% CO_2_ and 95% air. BRIN-BD11 cells were subsequently seeded into 96-well plates (5 × 10^3^ cells/well) or 24-well plates (1.5 × 10^5^ cells/well) and incubated overnight to allow cell attachment before being used for experiments. All reagents for cell culture were obtained from Sigma-Aldrich.

### 2.6. Acute and Chronic Insulin Secretion

Both acute and chronic insulin secretion measurements were performed as described by Rowlands et al. [26]. Briefly, to study the effect of amylase supplementation on chronic insulin secretion, BRIN-BD11 cells were incubated in their routine culture medium for 24 h in the absence or presence of amylase; then, the cell medium was collected for subsequent insulin measurements. Porcine pancreatic amylase (cat no. A3176, Sigma-Aldrich) was administered as a sterile solution in 0.9% NaCl, at final concentrations of 16, 32, or 64 *μ*g/ml, resulting in a final activity of 35.9, 69.2, and 114.5 U/l, respectively. Sterile 0.9% NaCl was used as a vehicle.

To detect possible effects of amylase supplementation on acute insulin secretion, the cells were starved for 40 min in a low-glucose Krebs-Ringer bicarbonate buffer, pH 7.4, 1.1 mM D-glucose (KRBB). Subsequently, stimulation for 20 min in KRBB, containing 16.7 mM D-glucose, plus 10 mM L-alanine (sKRBB), or in basal KRBB, containing 1.1 mM D-glucose (bKRBB), in the absence or presence of amylase was performed before medium collection. The final concentration of amylase was 32 *μ*g/ml (69.2 U/l), which theoretically corresponds to the amount of amylase released into the pancreatic interstitial fluid during one hour (ca. 60-70 U/l). Sterile 0.9% NaCl was used as a vehicle.

The insulin concentration in the medium was determined using an Ultrasensitive Rat Insulin ELISA kit (cat no. 10-1251-01, Mercodia). After removal of the medium, the cell layer was washed 3 times with PBS, and the cells were dried at room temperature and then dissolved in 0.2 M NaOH, for determination of the protein content according to Lowry et al. [27], using bovine serum albumin (BSA, Sigma cat no. A5470, Sigma-Aldrich) as the standard. The insulin content in the medium was normalized to the cellular protein content in the culture. The amylase activity in the medium was assessed as described above.

### 2.7. Statistical Analysis

Data is expressed as mean ± standarddeviation (SD). The distribution of the parameters was checked using a Shapiro-Wilk normality test. For *in vivo* experiments, the total area under the curve (AUC) was calculated for postload blood glucose, insulin, and glucagon levels. Blood glucose, amylase activity, and insulin and glucagon levels at different time points within one experimental group (with and without amylase) were compared using a paired Student *t*-test. As group variances were different, Brown-Forsythe and Welch ANOVA tests were used to assess statistical differences between groups. Data was not corrected for multiple comparisons. For *in vitro* data, a one-way ANOVA with a Tukey correction for multiple comparisons was used to estimate differences. In all statistical analyses, *p* ≤ 0.05 was considered significant. All analyses were carried out using Prism, version 8 (GraphPad Software, Inc., San Diego, CA, USA).

## 3. Results

### 3.1. In Vivo Studies

**Figure 2:**
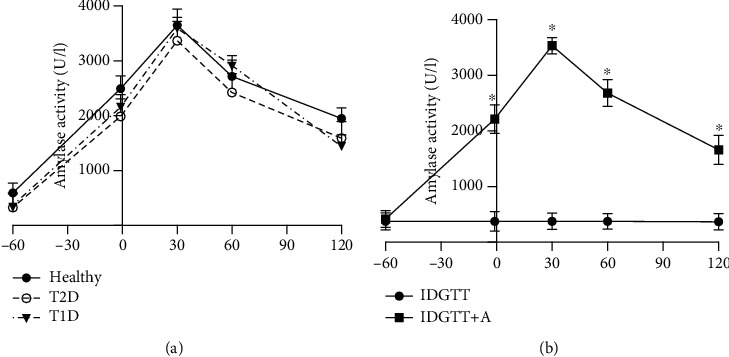
(a) Plasma amylase activity in healthy pigs (*n* = 4); pigs with STZ-induced type 2-like diabetes mellitus, which were administered STZ at 110 mg/kg bw (*n* = 4); and pigs with STZ-induced type 1-like diabetes mellitus, which were administered STZ at 150 mg/kg bw (*n* = 4) during an intraduodenal glucose tolerance test with an intravenous pancreatic amylase infusion (IDGTT+A). Data were analyzed using Brown-Forsythe and Welch ANOVA tests and are presented as mean ± SD. (b) Plasma amylase activity in experimental animals (*n* = 12), during an intraduodenal glucose tolerance test (IDGTT), either alone or together with an intravenous pancreatic amylase infusion (IDGTT+A). Data were analyzed using a paired Student *t*-test and are presented as mean ± SD. ∗ describes significant differences between IDGTT and IDGTT+A in corresponding time points when *p* < 0.05.

**Figure 3:**
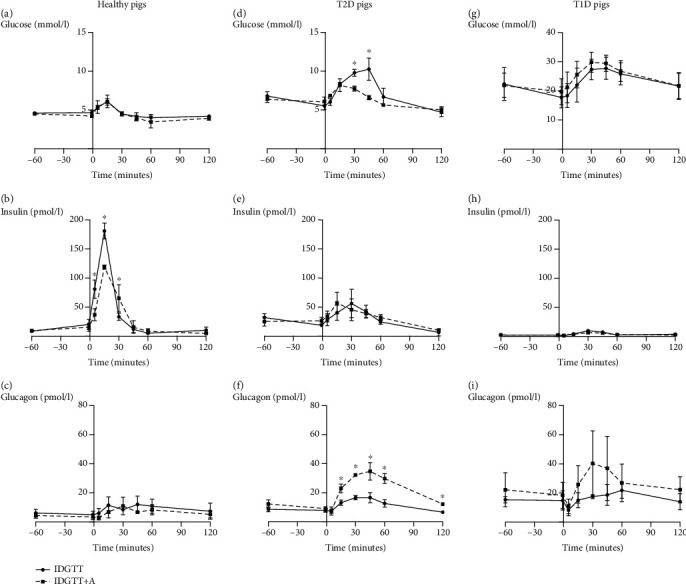
Plasma parameters in healthy pigs (*n* = 4) (a–c); pigs with STZ-induced type 2-like diabetes mellitus, which were administered STZ at 110 mg/kg bw (*n* = 4) (d–f); and pigs with STZ-induced type 1-like diabetes mellitus, which were administered STZ at 150 mg/kg bw (*n* = 4) (g–i). Blood glucose levels (a, d, g), plasma insulin (b, e, h), and glucagon concentrations (c, f, i) during an intraduodenal glucose tolerance test (IDGTT), either alone or together with an intravenous pancreatic amylase infusion (IDGTT+A). Data were analyzed using a paired Student *t*-test and are presented as mean ± SD. ∗ describes significant differences between IDGTT and IDGTT+A in corresponding time points when *p* < 0.05.

Basal levels of amylase activity and those following amylase intravenous infusion were not significantly different between groups (Figure 2(a)). An intravenous infusion of amylase to fasted pigs significantly increased blood amylase activity level (Figures 2(a) and 2(b)). Blood amylase levels were approximately 5.7-fold higher 1 hour following the first amylase infusion prior to the IDGTT, compared to baseline levels and compared to those observed at the corresponding time point without infusion of exogenous amylase (Figure 2(b)). The first intravenous infusion of amylase did not affect basal glucose, insulin, and glucagon levels in healthy, T2D, and T1D pigs, which were measured directly before the amylase infusion and one hour after, just prior to the IDGTT (Figures 3(a)–3(i)).

#### 3.1.1. Healthy Animals

Fasting blood glucose levels in healthy pigs were around 4.5 mmol/l (Table 1) and increased to 6.1 mmol/l following glucose infusion into the duodenum, after which a prompt reduction in blood glucose levels was noted. Fasting plasma insulin concentration in healthy pigs was approximately 8-10 pmol/l (Table 1). A significantly lowered increase in plasma insulin levels was observed at 5, 15, and 30 minutes after the intraduodenal glucose infusion (*p* = 0.003, 0.0001, and 0.039, respectively) following amylase pretreatment, compared to that observed during the control IDGTT, without amylase infusion (Figure 3(b)). Moreover, area under the curve (AUC) for insulin was significantly lower (*p* = 0.029) following amylase infusion (Table 1). Initial blood glucagon levels in healthy pigs were between 4 and 6 pmol/l and reached a peak of 11.5 pmol/l following glucose infusion into the duodenum; however, the glucagon release appeared to be unaffected in healthy animals by amylase pretreatment following the IDGTT (Table 1, Figure 3(c)).

#### 3.1.2. Pigs with T2D

In T2D pigs, fasting blood glucose levels were significantly (*p* = 0.002) higher than those observed in healthy pigs (Table 1) and were in the range of between 6.4 and 6.8 mmol/l. The first infusion of amylase prior to the IDGTT did not affect the pigs' state of euglycemia, similar to that observed in the healthy pigs (Figure 3(d)). Intraduodenal infusion of glucose during the control IDGTT in the T2D pigs resulted in a rapid increase in blood glucose concentration, up to 10.3 mmol/l at 45 min postinfusion (Figure 3(d)). Intravenous infusion of amylase directly prior to the IDGTT led to a significantly lowered increase in blood glucose level at 30- and 45-minute postinfusion (*p* < 0.001 and *p* = 0.002, respectively) was observed; moreover, this resulted in a significantly lower postinfusion glucose AUC (*p* = 0.007) (Table 1, Figure 3(d)) in T2D pigs. Fasting hyperinsulinemia was observed in T2D pigs, as their plasma insulin levels were between 20 and 30 pmol/l and were significantly higher (*p* = 0.03) when compared to that observed in the healthy pigs (Table 1). However, despite high initial values, insulin release AUCs in T2D pigs were not different from those observed in the healthy pigs and were unaffected by amylase pretreatment (Table 1, Figure 3(e)).

Initial glucagon levels in T2D pigs were higher when compared to those observed in the healthy pigs (*p* = 0.01) (Table 1). The intraduodenal glucose infusion in T2D pigs during the control IDGTT led to an increase in plasma glucagon concentration from 8.55 to 16.61 pmol/l, which was then followed by a reduction in glucagon concentration (Figure 3(f)). Amylase pretreatment resulted in a significantly larger increase in glucagon release, reaching 34.79 pmol/l at 45 min following the glucose infusion, with a corresponding 2.5-fold increase in the AUC (*p* < 0.0001) (Table 1, Figure 3(f)).

#### 3.1.3. Pigs with T1D

T1D pigs had fasting glucose levels in the range of between 17 and 26 mmol/l, which were significantly higher compared to values observed in both the healthy pigs (*p* = 0.008) and the T2D pigs (*p* = 0.01) (Table 1). The first infusion of amylase prior to the IDGTT did not influence the basal glucose level (Figure 3(g)). The IDGTT resulted in an increase in blood glucose concentration of up to 27.72 mmol/l. Amylase infusion had no effect on the glucose curve (Figure 3(g)). Despite extremely low plasma insulin levels in the T1D pigs, insulin release was stimulated by the intraduodenal infusion of glucose; however, it appeared to be unaffected by amylase pretreatment (Table 1, Figure 3(h)). The initial glucagon levels were significantly higher in the T1D pigs when compared to healthy and T2D pigs (*p* = 0.05) (Table 1). The intravenous amylase infusion in T1D pigs also resulted in a significant increase in glucagon release (*p* = 0.001 for glucagon AUC) following the amylase infusion during the IDGTT, compared to that obtained without amylase administration (Table 1, Figure 3(i)).

### 3.2. In Vitro Studies

**Figure 4:**
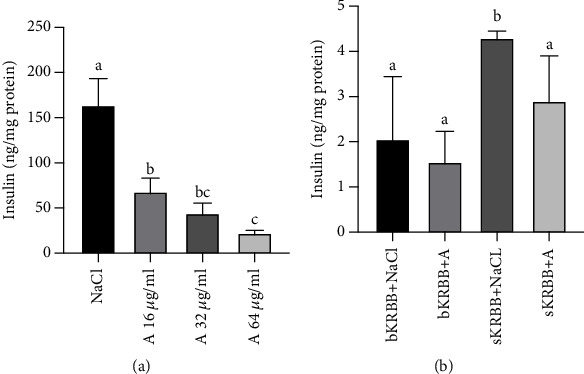
Insulin concentration in the media of BRIN-BD11 cells, after chronic (a) and acute (b) insulin secretion assay. (a) Chronic insulin secretion: 1 day after seeding of BRIN-BD11 cells in 96-well plates (5 × 10^3^ cells/well), the cells were incubated for another 24 h in their regular growth medium, in the absence or presence of porcine pancreatic amylase (a) (final concentrations of 16, 32, and 64 *μ*g/ml, resulting in a final activity of 35.9, 69.2, and 114.5 U/l, respectively). Sterile 0.9% NaCl was used as a vehicle. Data are the mean of 32 independent cell cultures (for each treatment) from two independent experiments. (b) Acute insulin secretion: BRIN-BD11 cells were seeded in 24-well plates (1.5 × 10^5^ cells/well) in the routine medium. After 24 h of incubation, the cells were starved for 40 minutes in bKRBB. The cells were then challenged with sKRBB, in the presence or absence of 32 *μ*g/ml porcine pancreatic amylase (a) for 20 min. Sterile 0.9% NaCl was used as a vehicle. Data are the mean of 12 independent cell cultures (for each treatment) from two independent experiments. bKRBB: basal medium; sKRBB: stimulative medium.

The Effect of Amylase on Acute and Chronic Insulin Secretion in BRIN-BD11 Cells. The insulin concentration in the culture media of BRIN-BD11 cells after 24 h of incubation with the vehicle (0.9% NaCl) was 164 ± 30 ng/mg protein. Addition of amylase resulted in a significant decrease in insulin concentration, which was dose-dependent. Thus, an amylase concentration of 16 *μ*g/ml led to a 2.6-fold decrease (*p* = 0.0484), and an amylase concentration of 32 *μ*g/ml resulted in a 3.7-fold decrease (*p* < 0.0001), while an amylase dose of 64 *μ*g/ml resulted in a 7.5-fold decreases in insulin levels (*p* < 0.0001) (Figure 4(a)).

With regard to the acute insulin secretion, the basal content of insulin in the basal medium (bKRBB) was 2.06 ± 1.38 ng/mg protein and it was not affected by the addition of amylase (Figure 4(b)). The stimulative medium (sKRBB) had a strong insulinotropic effect and stimulated a 2-fold increase in insulin levels, while the addition of amylase (sKRBB+A) significantly (*p* = 0.0127) reduced its insulinotropic effect by 1.5-fold, resulting in a decreased insulin concentration in the medium compared to that observed in the stimulative medium with vehicle (sKRBB+NaCl) (Figure 4(b)).

All the data were normalized to the cellular protein content in the cultures. Data were analyzed using a one-way ANOVA with a Tukey correction for multiple comparisons and are presented as mean ± SD. Different small letters given with result bars describe significant differences when *p* < 0.05.

## 4. Discussion

Our previous studies demonstrated that blood amylase participates in glucose metabolism and assimilation, reducing the glucose-stimulated insulin response in healthy, growing pigs and in pigs that have undergone bariatric surgery [23].

The present study revealed different effects of an intravenous amylase infusion on glucose absorption/metabolism and on glucagon/insulin release in healthy and in STZ-induced diabetic pigs, as well as direct effects of amylase supplementation on acute and chronic insulin secretion in BRIN-BD11 cells. The data obtained show that the intravenous infusion of STZ at a dose of 110 mg/kg bw resulted in the development of moderate hyperglycemia and hyperinsulinemia. The resultant condition observed resembled that which is often seen in people with early-stage type 2 diabetes mellitus [28, 29]. Koopmans et al. [15] described the development of hyperglycemia in a porcine model of STZ-induced diabetes mellitus as a fasting blood glucose level > 10 mmol/l, and the authors did not recognize a STZ dose of 110 mg/kg bw as a sufficient dose to induce a type 2 diabetes-like condition. However, our results show a 42% increase in fasting blood glucose levels in the pigs following the infusion of a low dose of STZ. In turn, a higher dose of STZ (150 mg/kg bw) infused intravenously led to a clear hyperglycemic and hypoinsulenemic diabetic state, which corresponds to that observed during type 1 diabetes mellitus [15]. Both T2D and T1D pigs demonstrated significant basal hyperglucagonemia. This finding is in agreement with that of Muller et al. [30], in which hyperglucagonemia was also observed in humans and animals with untreated type 1 diabetes.

The present study showed that the initial intravenous infusion of amylase, which took place one hour prior to glucose loading, did not affect the pigs' euglycemia, insulinemia, or glucagonemia (Figures 2(a)–2(i)). Amylase iv infusion directly prior to the IDGTT did however have various effects on the abovementioned parameters in the healthy, T2D, and T1D pigs. Previously, we suggested that amylase has possible regulatory effects only on postprandial hyperglycemia and on insulin/glucagon release, as in previous studies by our lab, we showed that the mode of amylase infusion did not affect basal glucose levels [23]. The same effect was also observed with regard to the acute secretion of insulin in the BRIN-BD 11 cell line, where basal insulin secretion remained unchanged following the addition of amylase; however, the glucose-stimulated insulin release was significantly suppressed in the presence of amylase. Experiments on chronic insulin secretion in the BRIN-BD 11 cells showed that 24 h treatment with amylase resulted in a decreased insulin concentration in the cell medium; thus, long-term stimulation of amylase secretion or treatment with amylase may possibly affect basal insulinemia *in vivo*. The direct effect of amylase on pancreatic beta-cells shown in the current study requires further detailed investigation; moreover, the possible effect of amylase on the alpha-cells of the pancreatic islets remains unclear.

Both *in vitro* and *in vivo* results described in the present study showed that amylase levels in both the cell medium and the blood affect the glucose-stimulated insulin response and these findings are in line with previous data from our lab [23]. Intravenous infusion of amylase did not affect the glucose curve in the group of healthy pigs; however, the insulin AUC after the IDGTT was significantly decreased. Acute glucose-stimulated insulin release in the BRIN-BD 11 cells was 1.5-fold lower in the presence of amylase in the cell medium. We can conclude that the effect observed *in vivo* was a result of the direct effect of plasma amylase on the beta-cells of the pancreatic islets, since the utilization of the same amount of glucose required less insulin in the healthy pigs in the presence of amylase in the blood stream. Glucagon release seemed to be unaffected.

With regard to the T2D pigs, an intriguing finding was observed. The glucose absorption curve was significantly lowered after amylase pretreatment, while the insulin curve remained unchanged, which is most probably due to the beta-cell dysfunction in these pigs, leading to a reduced ability for insulin secretion. At the same time, glucagon release following the IDGTT with the preceding amylase infusion was increased more than twofold, compared to that observed during control conditions. Similar changes in the glucagon curve were observed in the group of T1D pigs after intravenous amylase pretreatment; however, the glucose curve remained unchanged. Thus, the effect of amylase on the gut metabolism of glucose cannot be excluded and, in a state of extremely low insulin levels, is mediated via interactions with glucagon. The direct effect of amylase on pancreatic alpha-cells can also not be excluded. The latter statement in some way justifies the dose of amylase infused, which should be able to significantly increase the level of amylase in the interstitial fluid surrounding the pancreatic islets. Especially, since in pigs treated with STZ+alloxan, exocrine pancreas prandial secretion is ameliorated [31] and the amount of host amylase reaching the interstitial fluid and then the circulation could be lowered.

The role of blood amylase in the regulation of glucose metabolism in an insulin-dependent manner is rather obvious; however, the amylase-glucagon interaction is not clear and should be further investigated. In previous studies on animal models of type 1 diabetes, the hyperglucagonemia observed was directly coupled to the presence of hyperglycemia, the magnitude of which was decreased by the suppression of glucagon, while infusion of exogenous glucagon restored the hyperglycemia [32–34]. Our observations show that in STZ-induced type 1-like diabetes, the amylase-stimulated increase in glucagon release does not affect the hyperglycemia, while in STZ-induced type 2-like diabetes, intravenous infusion of amylase prior to the IDGTT leads to a simultaneous decrease in the glucose curve and increased glucagon release, in an insulin-independent manner. This observation requires further investigation. Theoretically, in type 2-like diabetes, the abovementioned phenomenon could probably be explained by the minimal passage of glucose from the gut to the blood, caused by the amylase infusion, which stimulates glucose consumption in the gut tissues and its conversion most probably to glycogen during hyperinsulinemia. The glycogen produced could in turn stimulate the release of glucagon, which results in the mobilization of tissue glucose from glycogen. In the T1DM state, the amylase infusion provoked a significant increase in glucagon release, causing maximal glucose mobilization with minimal glucose consumption by the gut and/or other tissues during the hypoinsulinemic state.

*The possible mechanisms involved in the effect of amylase on insulin/glucagon secretion in healthy and streptozotocin-induced diabetic pig models.* Intravenous infusion of amylase during an IDGTT in healthy animals clearly resulted in the significant lowering of insulin release, without any effect on the glucose utilization curve and glucagon release. Thus, one can suggest a protective or inhibitory or anti-incretin role of blood amylase on insulin secretion/production. It is logical to speculate that the stimulatory effect of enteral glucose can provoke not adequate, too high insulin release most probably via incretin's action. Thus, blood amylase plays a role as a modulator of blood homeostasis, an anti-incretin factor. Simply, blood amylase does not allow unnecessary overproduction of insulin, since an already 40% lower than normal secretion of insulin performs an adequate metabolic action and appropriately reduces blood glucose after enteral glucose loading (as shown in our studies). Why not protect the pancreatic islets from exhaustion and as a consequence protect against the first step towards diabetes development?

Another possible benefit with regard to the action of amylase is the possible prevention of the development of insulin resistance, which usually develops due to the unnecessarily high levels of insulin released during carbohydrate consumption—for review, see Pierzynowski et al. [35], where numerous recent papers are cited that describe an inverse relationship between blood amylase levels and obesity (diabetes type 2) in both children and adults. Zhuang et al. in their article from 2016 describe a positive correlation between serum amylase levels and pancreatic beta-cell function; however, the authors only discuss pathways of insulin-dependent amylase secretion, but not the dependency of insulin secretion or insulin sensitivity on amylase and the ability of amylase to modulate beta-cell function [22].

During the DT2 state, amylase infusion did not affect insulin release, most probably due to beta-cell dysfunction, leading to reduced insulin secretion ability. However, amylase loading significantly lowered the postloading blood glucose level in TD2 pigs. Date et al. in their studies confirmed the ability of amylase to downregulate GLUT2 and inhibit gut glucose absorption, which could be the reason for observed decrease in glucose absorption [36]. In addition, the blood level of glucagon increased significantly during glucose infusion. These data provoke several interesting questions and highlight the limitations of the studies. The main question is, could glucagon levels increase after amylase loading stimulates the secretion of insulin as suggested by Song et al. [37] and at the same time mobilize glucose from the glycogen depot? Another question that arises is, does amylase per se stimulate glycogen production, which in turn stimulates glucagon release? These questions need to be further explored in order to highlight or confirm the role of glucagon in diabetes development, since GLP1 is broadly used as a drug to stimulate insulin secretion, which in the long run could actually impair beta-cell function. Other possible explanations for the improved glucose tolerance test outcome in DT2 pigs include the insulin-like action of amylase on peripheral tissues and/or the improvement of insulin sensitivity by amylase.

Data from the DT1 animal model also prove that a low level of insulin (100 times lower than in healthy animals and 40 times lower than in DT2 animals) extraordinarily sensitizes glucagon release to blood amylase concentrations.

Considering all the data mentioned above, one can postulate that in T1D, exogenous insulin could be a good therapy. However, minimizing glucagon production during diabetes could be a good alternative to improve diabetes and prevent the development of insulin resistance. All of which could be improved by the ability of amylase to inhibit the entrance of glucose in to the systemic circulation, as was previously postulated in 2015 by Date et al. [36]. The action of amylase in the gut possibly stimulates the conversion of glucose to glycogen in hepatocytes, myocytes, and enterocytes or even stimulates bacterial glucose consumption?.

*Study limitations.* The limitations of our study undoubtfully should be mentioned. Firstly, it is a holistic observational study, which cannot clarify the pathways of amylase-insulin-glucagon interactions. Secondly, the intraduodenal glucose tolerance test used in our research could not reflect or determine any mechanisms of intrapancreatic relationships and the use of a euglycemic clamp would have improved the outcome. Direct measurements of insulin sensitivity in future studies could improve our understanding of intrapancreatic interactions. Thirdly, only the BRIN-BD11 cell line has been used to estimate the effects of amylase on insulin secretion *in vitro*, while using isolated human or porcine pancreatic islets would be preferable, since they allow for simultaneous modulation of hormonal responses and the investigation into the causality of interactions. However, to our knowledge, the number of studies on pancreatic acini–islet–acinar axis interactions is limited.

## 5. Conclusions

To summarize, the results of the current study can be interpreted in a number of ways. However, the results obtained showed that pancreatic amylase directly effects pancreatic beta-cells and actively participates in the maintenance of postprandial glucose homeostasis in healthy pigs, as well as in the type 2 diabetes mellitus porcine model. We observed disturbances in amylase-glucagon-insulin interactions with regard to glucose metabolism in the STZ-induced type 1-like diabetes model, which does not require or involve an insulin response, since insulin production is almost completely destroyed. The ability of blood amylase to participate in the metabolism of glucose in an insulin-independent manner is obvious; however, the amylase-glucagon interactions should be further investigated.

## Figures and Tables

**Table 1:** The fasting and postprandial blood levels of glucose, insulin, and glucagon in control and STZ-induced type 2 and type 1 diabetic pigs.

Parameters	Healthy		T2DM		T1DM	
	AMY-	AMY+	AMY-	AMY+	AMY-	AMY+
Fasting levels						
Glucose (mmol/l)	4.6 ± 0.1^a^	4.5 ± 0.1^a^	6.8 ± 0.6^b^	6.4 ± 0.4^b^	22.3 ± 5.6^c^	21.9 ± 4.2^c^
Insulin (pmol/l)	8.6 ± 1.0^a^	9.8 ± 1.5^a^	32.16.5^b^	25.3 ± 7.9^b^	2.5 ± 0.8^c^	2.6 ± 0.4^c^
Glucagon (pmol/l)	6.1 ± 2.5^ab^	4.4 ± 1.9^a^	8.6 ± 1.7^bc^	12.3 ± 2.7^cd^	15.3 ± 2.2^d^	22.2 ± 11.7^cd^
AUC						
Glucose (mmol/l/120 min)	93 ± 20^a^	88 ± 26^a^	328 ± 40^b^	222 ± 27^c^	1038 ± 281^d^	1257 ± 259^d^
Insulin (pmol/l/120 min)	4114 ± 359^a^	3378 ± 370^b^	3640 ± 394^ab^	4115 ± 439^ab^	478 ± 79^c^	394 ± 44^c^
Glucagon (pmol/l/120 min)	1106 ± 262^a^	719 ± 171^a^	820 ± 112^ab^	2404 ± 160^c^	1180 ± 325^ab^	2700 ± 739^c^

Control group: healthy pigs (*n* = 4); T2D: pigs with STZ-induced type 2-like diabetes mellitus, which were administered STZ at 110 mg/kg bw (*n* = 4); T1D: pigs with STZ-induced type 1-like diabetes, which were administered STZ at 150 mg/kg bw (*n* = 4). Data were analyzed using Brown-Forsythe and Welch ANOVA tests and are presented as mean ± SD. The different small letters given with particular results in a row describe statistical significant differences when *p* < 0.05.

## Data Availability

All data relevant to the study are included in the article.
